# Introducing article-processing charges and inviting "detailed methods sections" articles

**DOI:** 10.1186/1742-5573-2-5

**Published:** 2005-06-07

**Authors:** Carl V Phillips

**Affiliations:** 1 Department of Public Health Sciences, University of Alberta, 13–103 Clinical Sciences Building, Edmonton, Alberta T6G 2G3, Canada

## Abstract

This editorial introduces the use of article-processing charges at *Epidemiologic Perspectives & Innovations *and reviews that advantages of publishing in an Open Access journal. In addition, it introduces a new type of article the journal hopes to publish, detailed reports of study design or data analysis methods that have been used in health science research. The new type of article is intended to supplement the woefully constrained methods sections in standard research report articles, providing information that better fulfills the goals of scientific publishing.

## Introduction

*Epidemiologic Perspectives & Innovations *(*EP&I*) is published by BioMed Central (BMC), an independent publisher committed to ensuring peer-reviewed research is Open Access – that it is universally and freely available online to everyone and its authors retain copyright. In order to fund the publication of the journal while fulfilling our commitment to make all content of *EP&I *free to readers, the publisher and editors of the journal have introduced an article-processing charge. Starting April 2005, authors of articles accepted for publication will be asked to pay a charge of £330 (currently approximately US$625 or €480). There is no charge if a submission is rejected. Authors at the many BMC member institutions receive an exemption from the fee, and we encourage authors to help persuade their institutions to become members if they are not already (see endnote 1). Waiver requests, particularly from authors with financial hardship, will be considered on a case-by-case basis.

For paper journals, the standard subscription model is that readers pay, either through personal or institutional subscriptions, and this is typically extended to online versions. The result is that many potential readers are discouraged from viewing articles. Libraries, faced with escalating journal costs, have had to cut back on their commitment to providing access. Moreover, except for the shortest articles, authors at many paper journals have to pay a per-page fee. Unlike these page charges, the BMC article processing charge is a flat fee, regardless of the length of the article, accompanying graphics, or attached files, and thus is relatively modest.

## Advantages of Open Access

*EP&I*'s policy of Open Access changes the way in which articles are published. All articles are available for free to anyone with internet access. Readers are not limited by what their library can afford, and can easily access articles via web-based searches (using research databases or general web search engines), increasingly the most popular method for finding publications. This easier availability has been shown to make articles more highly cited [1]. It also fulfills the requirements that are increasingly being imposed by funders to make the products of their funding publicly available.

Under the Open Access model, authors retain copyrights to their work (beyond granting the journal the right to publish and archive the article, and readers the right to make appropriate use of the published material), allowing the authors to freely distribute, anthologize, and repost their work. This helps avoid such absurdities of the standard publishing model as authors having to pay substantial reprint fees (or violate copyright) to hand copies of articles to their students, often after already paying the journal to publish the article (see endnote 2).

Articles published at *EP&I *are available at the journal's website in addition to being archived in PubMed Central [2], the US National Library of Medicine's full-text repository of life science literature, and also in repositories at the University of Potsdam [3] in Germany, at INIST [4] in France and in e-Depot [5], the National Library of the Netherlands' digital archive of all electronic publications. Authors can easily check the number of times their article has been accessed via the *EP&I *website (though not the other archives), providing an estimate of the article's readership, another feature not possible with paper journal articles.

Increasingly, traditional paper journals are adopting certain Open Access policies piecemeal, including free online access (though often only part of their archives), online supplemental material, and article-processing charges. But by publishing at *EP&I *and other fully Open Access journals, authors can be assured of having all the advantages of Open Access: free access to all readers from the day of publication, retained copyright, and seamless online links to their articles and accompanying files, while retaining all the advantages of peer-review and appearance in scientific indexes.

## Publishing "better methods sections" articles

The online publishing model, combined with the mission of *EP&I*, allows us to provide a forum for articles that are unlikely to be published in paper journals [6]. We are pleased to have made a good start on our mission of broadening what is published as legitimate health science research. We have published or expect to publish soon articles on quantitative methodology, decision making, new software, teaching methods, historical perspectives, and re-analyses of previous results. Several of these, despite their very high quality, were unlikely to have been published in other health science journals. We would now like to further take advantage of online publishing and invite submissions of a new kind of article.

A *raison d'etre *of scientific publishing is to allow other researchers to assess the validity of a study and to replicate it by following the recipe themselves, goals that are not compatible with squeezing the methods into a thousand words, as is typically required. Sufficiently describing a field study's data collection methods seems to require at least three or four thousand words, as does all but the simplest data analysis (see endnote 3). A recent editorial in the *American Journal of Epidemiology *[8] went so far as to praise the authors of that issue's lead research article [9] for getting the article down to 2,164 words. We will resist the temptation to itemize what was noticeably missing from the 548-word methods section in the praised article (let alone speculate about important information that a reader might never even realize was omitted), and instead take the high road, offering a new outlet for researchers who are pressured to reduce the length of their articles in paper journals (see endnote 4).

*EP&I *will publish methods articles that are primarily descriptive, presenting the detailed workings of the design and implementation of a study, how the data was analyzed, or both. We welcome submissions that report in detail the methods that produced previously published results, or details of methods in advance of the publication of results. Such submissions should use the Methods article type (see our instructions to authors for details) and the title should either lead with "Detailed Methods:..." or otherwise include the phrase "detailed methods". Authors are welcome to report some study results to provide context, but the article's focus should be the methods, not the results. We also encourage investigative reporting – submissions by authors who have been able to determine some of the not-publicly-available details of the methodology used in influential research studies published by other authors (see endnote 5).

Since the purpose of this type of article is to provide enough detail to scrutinize or replicate a method, authors should include any detail that required a decision about study protocol or analysis method. Though we have no set limit, we would expect any submission that contained enough detail to be more than 4000 words long. Details that should be reported include:

• What previously established study designs were used as a template for the study and why? What variations on that template were introduced, and why?

• What data analysis methods (specific statistical methods, choices of functional forms, subgroup analyses, etc.) were tried and what results did they produce? Why was a certain one chosen to produce "the" results of the study? If results have been published, what other data analyses were run but not reported, and why?

• Why were certain cutpoints chosen to categorize variables, define exclusion criteria, etc.?

• What design decisions, in retrospect, were suboptimal or led to notable problems? What changes in the originally conceived protocol were introduced and why?

• What, if anything, in the study design or analysis method is particularly noteworthy and recommended for use to future researchers?

To better conceptualize the goals of such publications, authors should think of themselves as trying to fulfill one or more of the following goals:

• Providing enough detail about data sources and analysis to allow readers to make better use of the study results. Such uses could include quantifying uncertainty about the study results (see, e.g., [10-13]), incorporating the results into meta-analysis or other summary analysis, or assessing the basic validity of the conclusions and the degree of "publication bias in situ" [7]. Typically, readers must guess at such details, dramatically diminishing the value of the study to the science.

• Presenting a case-study lesson for researchers who want to learn more about data gathering and analysis methods. Typically junior health researchers learn methods from one mentor or the overly-idealized presentations in textbooks, and even senior researchers have limited information about how others do things. There is a lot of room for better open exchange.

• Telling the story of how health science actually happens, as might be of interest to such chroniclers as historians of science, sociologists, and popular science writers.

Some partial solutions to the problem of diminutive methods sections already exist, including online methodology appendices and the reporting of a study's methods across multiple published articles. But these options have limitations and do not go as far as our proposed article type. Moreover, they do not properly reward authors with credit for a publication for the extra effort required to report their methods in a scientifically complete manner.

## Conclusion

We are delighted to be able to provide this forum for scientific publishing and to be able to participate in the Open Access movement. We hope you will support this effort by submitting a paper to *EP&I *and submitting your reports of study results to other Open Access journals.

## Endnotes

1. For a list of member institutions, see .

2. For more details on copyright and other policies, see the BioMed Central Open Access Charter, .

3. The value of carefully reported methods, rather than broad sketches, is exemplified by the huge number of choices that must be made about how data is analyzed and reported, and the dramatic effect those choices can have on the reported results [7].

4. We cannot resist pointing out a few observations: The length of the methods section of the praised article, which describes a field study and data analysis methods, comes out to about 70 words per author. The article's discussion section is almost twice as long as the methods, and though our journal encourages articles that are entirely "discussion" [6], this seems imbalanced for a report of study results. The editorial also went so far as to suggest that articles over 3000 words are "often boring." Regarding the latter point, we believe that readers of *EP&I *will find ample evidence to the contrary.

5. Such information might be inferred by replicating the results, examining regulatory or litigation testimony, or other methods. We encourage authors of such analyses to contact the original study authors to confirm or add to the analysis, and to invite them to be coauthors if they are willing to contribute substantially.

## Competing interests

Parts of this editorial were adapted from a previous article (Slade E et al. *Critical Care *2003, 7:331–332) that was suggested as a template by the publisher (used by permission).

Some critics of the author-pays publishing model have suggested that journals have a financial incentive to accept more articles to increase revenue, allowing authors to buy their way into publications. We believe that the quality of articles in *EP&I *speaks for itself, and makes it clear that our decisions to publish are based on the quality of the work. At this point, the entire article-publishing charge goes to the publisher, not to the editorial side of the journal (which makes decisions about what to publish). All scientific researchers and journal editors have motives outside some Platonic ideal of scientific publishing. These include the oft-cited financial interests, but perhaps more importantly also the bolstering of careers (publishing more articles thickens the CVs of authors, and increases the credit we get for being editors) and scientific politics (health researchers are very often trying to confirm something they believe to be true – regardless of who funded the study! – and the editors of this journal are trying to change how health research is done and reported). We continue to encourage authors to disclose competing interests, and to not mistake that phrase for "funding source".
